# Regulation of PDGFC signalling and extracellular matrix composition by FREM1 in mice

**DOI:** 10.1242/dmm.013748

**Published:** 2013-08-15

**Authors:** Fenny Wiradjaja, Denny L. Cottle, Lynelle Jones, Ian Smyth

**Affiliations:** 1 Department of Biochemistry and Molecular Biology, Monash University, Wellington Road, Clayton, Victoria 3800, Australia; 2 Department of Anatomy and Developmental Biology, Monash University, Wellington Road, Clayton, Victoria 3800, Australia

## Abstract

Fras1-related extracellular matrix protein 1 (FREM1) is required for epidermal adhesion during embryogenesis, and mice lacking the gene develop fetal skin blisters and a range of other developmental defects. Mutations in members of the *FRAS/FREM* gene family cause diseases of the Fraser syndrome spectrum. Embryonic epidermal blistering is also observed in mice lacking *PdgfC* and its receptor, PDGFRα. In this article, we show that FREM1 binds to PDGFC and that this interaction regulates signalling downstream of PDGFRα. Fibroblasts from *Frem1*-mutant mice respond to PDGFC stimulation, but with a shorter duration and amplitude than do wild-type cells. Significantly, PDGFC-stimulated expression of the metalloproteinase inhibitor *Timp1* is reduced in cells with *Frem1* mutations, leading to reduced basement membrane collagen I deposition. These results show that the physical interaction of FREM1 with PDGFC can regulate remodelling of the extracellular matrix downstream of PDGFRα. We propose that loss of FREM1 function promotes epidermal blistering in Fraser syndrome as a consequence of reduced PDGFC activity, in addition to its stabilising role in the basement membrane.

## INTRODUCTION

The FRAS/FREM extracellular matrix (ECM) proteins (FRAS1, FREM1 and FREM2) mediate adhesion between the epidermal basement membrane and the underlying dermis during embryonic development (reviewed in Short et al., 2007; Petrou et al., 2008). Consequently, their mutation results in the formation of fetal epidermal blisters in a number of human conditions, including Fraser syndrome (FS; *FREM2* and *FRAS1* mutations), Manitoba oculotrichoanal syndrome (MOTA) and bifid nose/anorectal and renal anomalies syndrome (BNAR) (both caused by mutations in *FREM1*) (McGregor et al., 2003; Vrontou et al., 2003; Jadeja et al., 2005; Alazami et al., 2009; Vissers et al., 2011). These conditions encompass a wide range of developmental defects, of which cryptophthalmos, syndactyly, renal agenesis, ambiguous genitalia and respiratory tract defects are prominent. The FS-spectrum diseases are phenocopied by the ‘blebs’ family of mouse mutants, whose causative mutations lie in the mouse homologues of these FRAS/FREM genes (McGregor et al., 2003; Vrontou et al., 2003; Smyth et al., 2004; Jadeja et al., 2005). FRAS1 and FREM2 are expressed exclusively by epidermal cells, whereas FREM1 is expressed in both the dermis and epidermis (Petrou et al., 2007; Short et al., 2007). FRAS1, FREM1 and FREM2 are then thought to interact in a complex to stabilize and cross-link epidermal basement membrane attachment to the developing dermis (Kiyozumi et al., 2006).

The FRAS/FREM family of proteins share characteristic chondroitin sulphate proteoglycan (CSPG) core repeats similar to those found in the NG2 proteoglycan (Stallcup, 2002). In this protein, they directly bind platelet-derived growth factor A (PDGFA), fibroblast growth factor FGF2, and collagens V and VI (Goretzki et al., 1999). Epidermal blistering defects like those observed in FS syndrome and the blebs mice are also observed in mice lacking either the growth factor PDGFC or its receptor, PDGFRα (Soriano, 1997; Tallquist and Soriano, 2003). PDGFC is expressed in a number of developing epithelia, including the epidermis, with complementary expression of PDGFRα observed in the associated underlying mesenchyme (Ding et al., 2000; Aase et al., 2002). PDGFC signalling acts upstream to drive expression of matrix metalloproteinase-1 (MMP1) and its inhibitor, tissue metalloproteinase inhibitor 1 (TIMP1), *in vitro* and in transgenic overexpression models (Campbell et al., 2005; Jinnin et al., 2005).

Based on the observation that CSPG domains can interact with PDGF proteins and that epidermal blistering caused by *Fras/Frem* gene mutations in developing blebs mice is ultrastructurally, spatially and temporally similar to that observed in *PdgfC* mutants, we hypothesised that FREM1 might regulate the capacity of PDGFC to regulate downstream remodelling of the extracellular matrix (ECM). We show that FREM1 binds to PDGFC *in vitro* and *in vivo* and demonstrate in mouse embryonic fibroblasts (MEFs) that wild-type (WT) FREM1 maintains the normal duration and amplitude of PI3-kinase (PI3K)/AKT and MAPK activation following PDGFC stimulation. We further demonstrate that this interaction regulates expression of metalloproteinase inhibitor *Timp1* and collagen I deposition. We therefore propose that FREM1 potentiates PDGFC signalling, which in turn shapes ECM processing and composition during development. These observations provide a mechanistic basis for basement membrane fragility that leads to epidermal blistering in FS-spectrum diseases and in the blebs mutant mice that model them.

**TRANSLATIONAL IMPACT**

**Clinical issue**

Fraser syndrome (FS), an autosomal recessive developmental disorder, is caused by mutations in members of the FRAS and FREM family of extracellular matrix (ECM) proteins. The FRAS and FREM proteins are thought to function collectively and cooperatively to structurally cross-link components of the basement membrane in developing epithelia. FRAS and FREM mutations found in individuals with FS (or in the *blebs* family of mouse mutants that phenocopy the disorder) lead to defects in epidermal adhesion and the formation of large skin blisters *in utero*. These blisters are thought to contribute to the formation of a number of craniofacial and soft tissue malformations, including the signature feature of FS, cryptophthalmos, in which skin covers the globe of the eye. Based on earlier studies conducted in mice, it was noted that mutation of FREM1, one of the key proteins implicated in FS, leads to phenotypes that paralleled those caused by loss of platelet-derived growth factor receptor alpha (PDGFRα) signalling, and might therefore control ECM remodelling. However, this hypothesis has not been directly tested.

**Results**

The authors focussed on PDGFC, a key component of the PDGFRα pathway. Using immunoprecipitation, cell culture and transfection experiments in a mutant mouse line, they show that FREM1 binds to PDGFC *in vitro* and *in vivo*. They show that FREM1 colocalises with PDGFC in the basement membrane, in close proximity to PDGFRα in dermal fibroblasts. Furthermore, they demonstrate that the interaction between PDGFC and FREM1 potentiates signalling via PDGFRα. This signalling cascade controls expression of the matrix metalloproteinase inhibitor TIMP1, which in turns plays an active developmental role in regulating ECM deposition, including that of collagen I, part of the foundation of the basement membrane.

**Implications and future directions**

This study provides some of the first evidence that FREM proteins bind to additional ECM factors in the extracellular milieu. By establishing a link between FREM1, PDGFC growth factor activity and the downstream regulation of molecules that shape ECM remodelling, the authors bring to light a novel mechanism for the development of basement membrane fragility and blistering in Fraser syndrome and its related diseases. Further studies that explore the interactions between the FREM and FRAS proteins and PDGFC or other ECM components will lead to a better understanding of these diseases, and could enable the identification of therapeutic targets.

## RESULTS

### FREM1 interacts with PDGFC

**Fig. 1.**
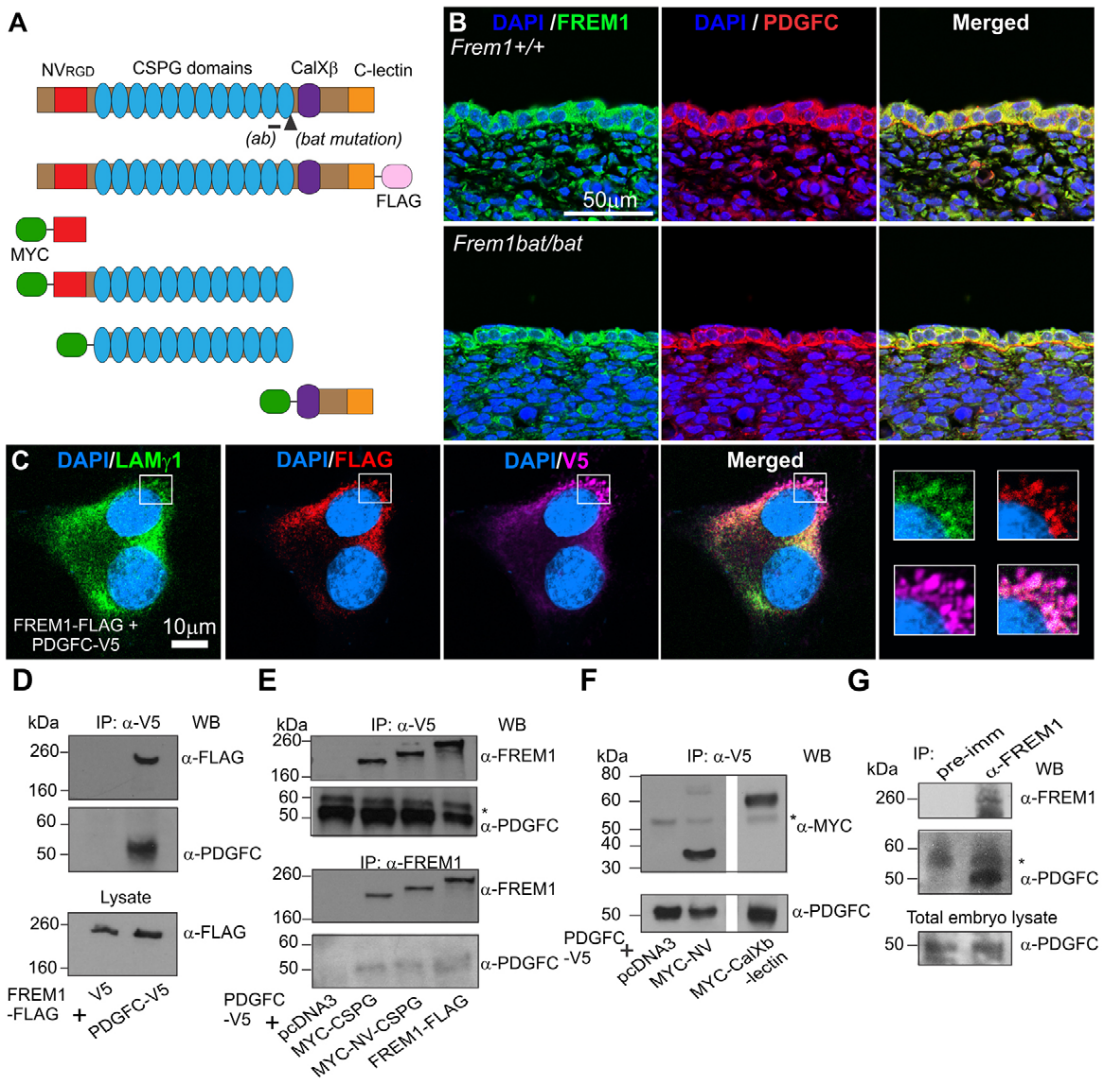
**Interaction between FREM1 and PDGFC.** (A) Structural representations of the full-length FREM1, FLAG-tagged construct and truncated constructs: NV alone, NV-CSPG, CSPG and CalXβ-C-lectin domains are shown with the tags indicated. The MYC tag shown refers to a 3×MYC tag + Igκ secretion signal. (*ab*), location of immunising sequence for anti-FREM1 antibodies upstream of mutation. (B) E13.5 WT and *bat* embryo head skin sections stained for FREM1 (green), PDGFC (red) and nuclear dye DAPI (blue). (C) NIH3T3 fibroblasts expressing FREM1-FLAG and PDGFC-V5 and immunostained as indicated. (D) Coimmunoprecipitation of FREM1-FLAG and PDGFC-V5 in transfected HEK293 cells. (E,F) Co-immunoprecipitation of PDGFC-V5 with MYC-tagged FREM1 subdomains. (G) Co-immunoprecipitation of endogenous FREM1 and PDGFC from embryo extracts at E12.5. A rabbit pre-immune serum was included as a control. * IgG heavy chain; IP, immunoprecipitation antibody; WB, western blotting antibody.

FREM1 is a multi-domain protein and, in mice that are homozygous for the FREM1 *bat* mutation, a single DNA base change abolishes an intron splicing site. This aberrant intron inclusion leads to a frame shift and premature stop codon within the twelfth CSPG domain, thereby removing the C-terminal CalXβ and C-lectin domains (Smyth et al., 2004) (Fig. 1A). FREM1 expression has been previously established in both epidermal and dermal cells, and localises to the basement membrane separating the two populations (Petrou et al., 2007; Short et al., 2007). PDGFC is expressed by epidermal cells and diffuses to the underlying mesenchyme (Ding et al., 2000; Aase et al., 2002). To determine whether PDGFC and FREM1 colocalise, paraffin head skin tissue sections from E13.5 embryos were immunostained with rat anti-FREM1 and anti-PDGFC antibodies. We observed FREM1 localisation in the epidermis and basement membrane as previously reported, but additionally saw FREM1 in the ECM surrounding dermal fibroblasts (Fig. 1B). FREM1 *bat* mutant mice showed no changes in expression or localization of the FREM1 mutant protein. Immunostaining with rat non-immune antibody was performed as a control and confirmed the specificity of the rat anti-FREM1 signal (data not shown).

Consistent with reported literature, we also observed PDGFC enriched in the epidermis and weakly in the dermis; however, most significantly, we observed PDGFC on the basement membrane (Fig. 1B). Thus, there is substantial overlap in the localisation of FREM1 and PDGFC within keratinocytes, on the basement membrane and within local ECM surrounding dermal fibroblasts. We did not see a change in the distribution of PDGFC in *bat* mice (Fig. 1B).

To further explore potential interactions between the proteins, NIH3T3 cells were transfected with plasmid constructs encoding full-length FREM1-FLAG (Fig. 1A) and PDGFC-V5. Detection of extracellular deposits of either factor was rare; however, FREM1-FLAG localised with secretory vesicles marked by the ECM component laminin γ1. PDGFC-V5 showed a broad intracellular distribution but was also enriched in laminin-γ1-positive vesicles, where it colocalised with FREM1-FLAG (Fig. 1C, see white boxes). This indicates that FREM1 and PDGFC can colocalise intracellularly, as well as in ECM components in the absence of a basement membrane *in vitro.*

To test for a physical interaction between FREM1 and PDGFC, HEK293 cells were transfected with constructs encoding full-length FREM1-FLAG and PDGFC-V5, or empty vector controls. Immunoprecipitation using a mouse V5 antibody revealed the presence of FREM1-FLAG in the PDGFC-V5 immunoprecipitate, but not in the V5 control pull-down (Fig. 1D). FREM1-FLAG and PDGFC-V5 ran as a 260-kDa and 55-kDa species, respectively, as demonstrated in earlier studies (Li et al., 2000; Kiyozumi et al., 2006). To determine which domain mediates binding to PDGFC, MYC-tagged FREM1 sub-domains (outlined in Fig. 1A) were co-expressed with PDGFC-V5. Interactions with NV, CSPG, NV-CSPG and CalXβ-C-lectin domains were identified (Fig. 1E,F). We consistently observed an ∼75 kDa band in the NV domain samples, suggesting potential dimerisation of the NV domain. Using the rabbit anti-FREM1 polyclonal antibody, we performed reciprocal experiments to pull down full-length FREM1, CSPG or NV-CSPG domains and detected PDGFC in all immunoprecipitates except the empty vector (negative control) (Fig. 1E). To confirm the physiological significance of these interactions, we coimmunoprecipitated FREM1 and PDGFC from embryonic protein extracts (Fig. 1G). Co-immunoprecipitation experiments using the unrelated microtubule-associated E3 ubiquitin ligase MID1, tagged with GFP (Short and Cox, 2006), were undertaken as negative controls (data not shown). Collectively this data demonstrates that FREM1 and PDGFC can colocalise and physically interact through multiple domains, in a physiologically relevant manner.

Full-length PDGFC exists in a latent form from which the N-terminal CUB domain is cleaved by plasmin or tissue plasminogen activator (tPA) to release the catalytic growth factor domain (GFD) (Li et al., 2000; Fredriksson et al., 2004). To further explore how FREM1 might affect PDGFC, we next examined processing of PDGFC. However, conditioned media from cells co-expressing tPA, PDGFC-V5 and FREM1-FLAG did not have increased levels of GFD (data not shown), suggesting that FREM1 does not affect processing. Because FREM1 influences the cellular response to the mature processed PDGFCC (the homodimer of PDGFC) when supplied in recombinant form (see below), we propose that the functional interaction between the proteins occurs after processing and dimerisation.

### FREM1 augments PDGFC signalling through PDGFRα

**Fig. 2.**
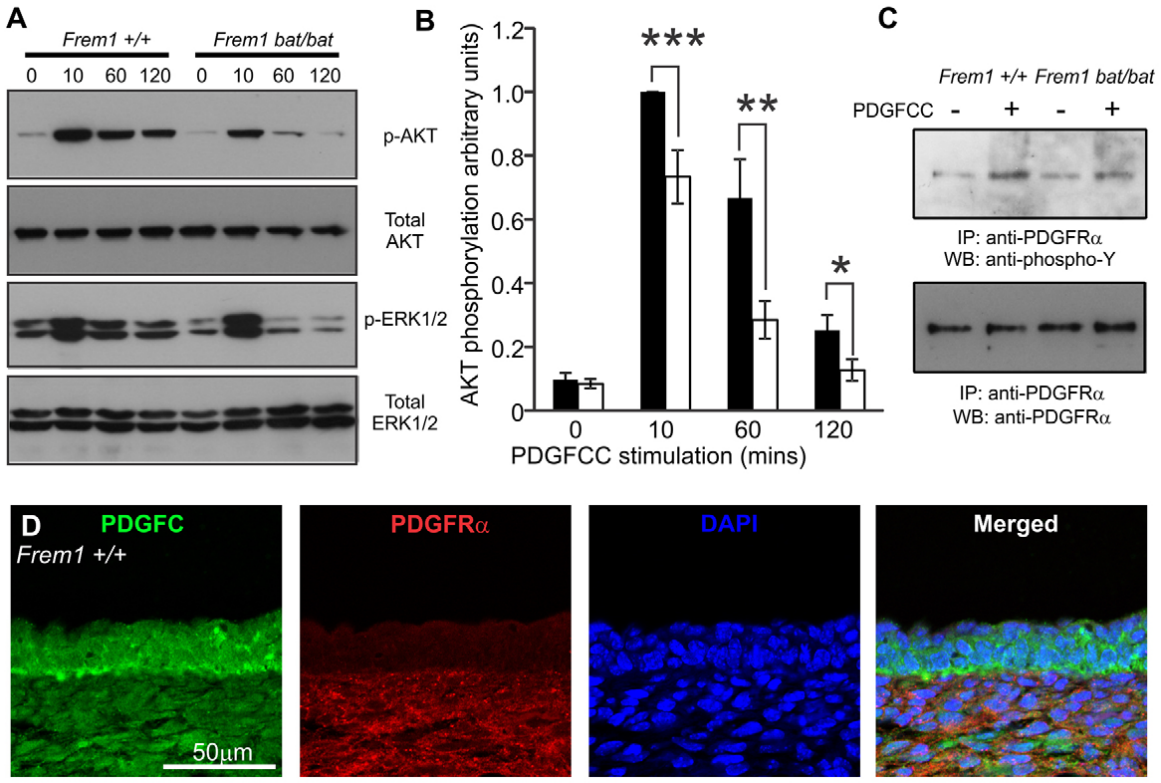
**FREM1 regulates activation of AKT and MAPK upon PDGFC stimulation.** (A) Representative western blotting of phosphorylation of AKT and MAPK ERK1/2 in MEFs from WT and *bat* mouse embryos stimulated with PDGFCC for the indicated time periods. (B) Relative quantification of AKT phosphorylation levels. WT cells 10 minutes after stimulation were assigned a value of 1 and all other samples are standardised against this value. Graph represents average of up to nine WT and 16 *bat* samples, performed across four independent experiments from at least three different cell lines for each genotype. Black bars: WT; white bars, *bat* mutant. (C) FREM1 mutation in *bat* mutants reduces phosphorylation of PDGFRα in response to the addition of exogenous PDGFCC. IP, immunoprecipitation antibody; WB, western blotting antibody. (D) E13.5 WT embryo head skin sections stained for PDGFC (green), PDGFRα (red) and nuclear dye DAPI (blue). Error bars represent standard error of the mean (s.e.m.); *P<0.05, **P<0.01, ***P<0.005.

The interaction of NG2 and PDGFA is thought to regulate signalling downstream of PDGFRα (Grako and Stallcup, 1995). To investigate the significance of FREM1-PDGFC interactions in a similar context, we profiled analogous receptor activation. MEFs form an attractive *in vitro* model because they originate from mesenchyme, secrete ECM and express endogenous FREM1 and PDGFRα, analogous to dermal fibroblasts. Binding of PDGFCC to PDGFRα stimulates auto-phosphorylation of the receptor, which leads to signalling through PI3K and MAPK activation pathways. Although stimulation of serum-starved MEFs derived from either *Frem1^+/+^* or *Frem1^bat/bat^* embryos for 10 minutes with 100 ng/ml PDGFCC resulted in upregulation of AKT phosphorylation, this was dramatically reduced in *Frem1^bat/bat^* cells compared with WT after 10, 60 or 120 minutes of stimulation (Fig. 2A,B). This difference was also reflected in ERK1/2 activation. Similar experiments using epidermal growth factor (EGF) as an agonist did not result in any differences in AKT or ERK1/2 phosphorylation between different genotypes (results not shown).

We wondered whether the overall decrease in AKT activation upon PDGFCC stimulation in *Frem1^bat/bat^* cells is due to impaired PDGFRα activation. MEFs were therefore stimulated with PDGFCC for 60 minutes and the levels of PDGFRα phosphorylation analysed by first immunoprecipitating total PDGFRα then immunoblotting with a phospho-specific antibody. We observed a reduction in phosphorylated PDGFRα in PDGFCC-stimulated *Frem1^bat/bat^* MEFs compared with WT cells when adjusted to total PDGFRα levels (Fig. 2C). To determine where the PDGFC and FREM1 interaction could potentially regulate a PDGF signal, paraffin head skin tissue sections from WT E13.5 embryos were immunostained for PDGFC and PDGFRα (Fig. 2D). PDGFRα was detected in dermal fibroblasts as published previously (Collins et al., 2011) and was strongest on their outer surface, overlapping with PDGFC in the dermis. Because FREM1 and PDGFC also colocalise here, the interaction might influence PDGFRα signalling globally throughout the dermis. However, the dermal fibroblasts immediately underlying the basement membrane are those most exposed to PDGFCC produced in the epidermis and hence are the most likely to be influenced by the association of FREM1 and PDGFC. The enrichment of all factors on the outside or outer surface of fibroblasts suggests that FREM1 might be involved in facilitating the presentation of PDGFC to PDGFRα.

### FREM1 regulates Timp1 transcription downstream of PDGFCC through a mechanism dependent on PDGFRα, PI3K and MAPK activation

**Fig. 3.**
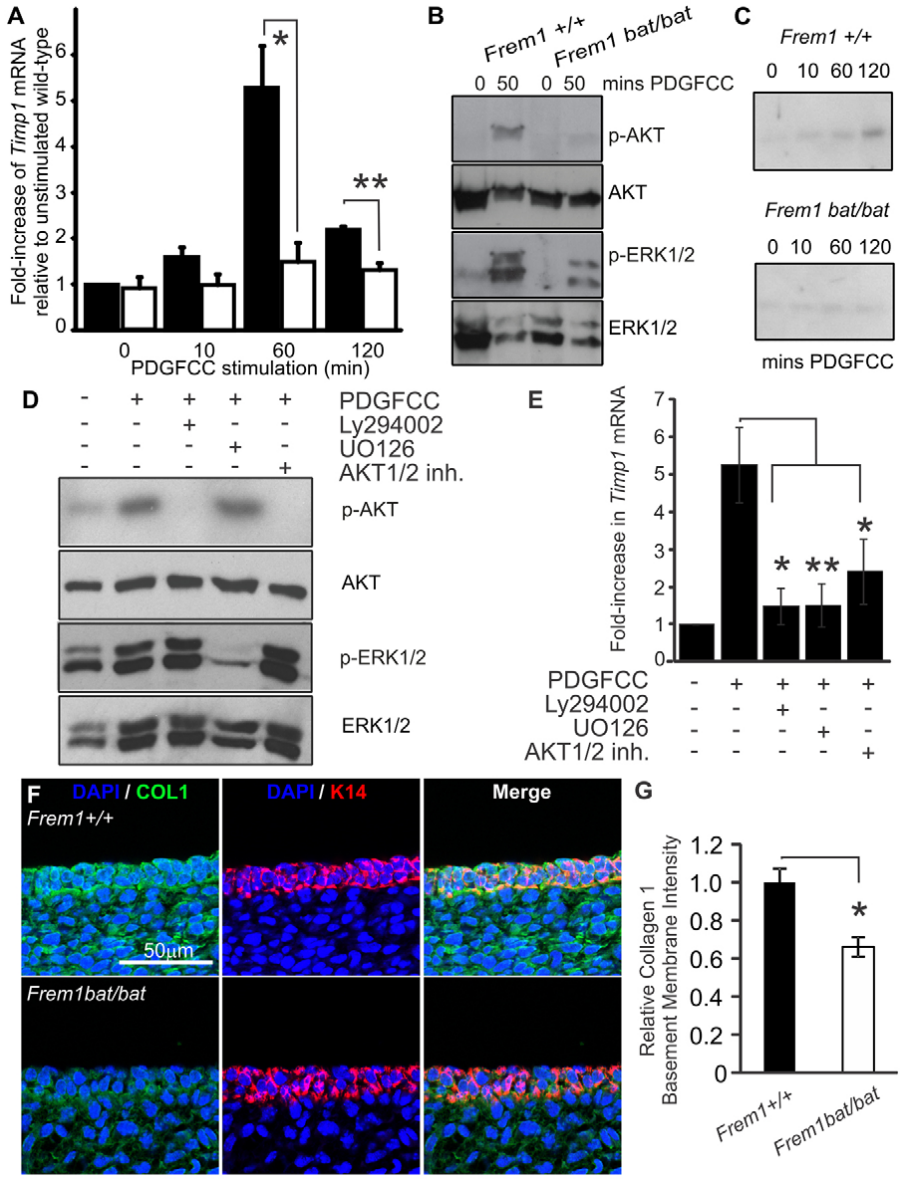
**FREM1 regulates *Timp1* transcription downstream of PDGFCC, through a mechanism that is dependent on PDGFRα, PI3K and MAPK activation.** (A) qRT-PCR analysis of *Timp1* mRNA levels in WT (black bars) and *bat* mutant (white bars) MEFs following PDGFCC stimulation for the indicated time points. Data was obtained from four independent experiments from at least three different cell lines for each genotype, and presented as fold increase relative to unstimulated WT cells. (B) Alterations in phosphorylation of AKT and ERK1/2 precede changes in *Timp1* expression. (C) TIMP1 protein secretion is reduced in stimulated FREM1 *bat* mutant MEF cultures. (D) WT cells were pre-treated with either the PI3K inhibitor LY294002, AKT1/2 inhibitor, MAPK inhibitor UO126 or DMSO vehicle control prior to PDGFCC stimulation and analysed by immunoblotting with anti-phospho-AKT, anti-phospho-ERK1/2, total AKT or total ERK1/2. (E) qRT-PCR for *Timp1* mRNA was performed on the same cells. Levels of *Timp1* mRNA were presented as a fold increase relative to unstimulated cells. Experiments were performed three times using three different cell lines. (F) E13.5 WT and *bat* embryo head skin sections stained for COL1 (green), keratin 14 (K14; red) and nuclear dye DAPI (blue). Error bars represent s.e.m.; *P<0.05, **P<0.01.

In addition to its role as a mitogen, numerous studies suggest that PDGFC controls the composition of the ECM in a number of developmental and disease contexts. In particular, in human dermal fibroblasts, PDGFCC stimulation induces the synthesis and secretion of TIMP1 (Jinnin et al., 2005), which is a key regulator of ECM processing and of basement membrane composition (reviewed in Singer and Clark, 1999). To investigate whether the interaction between FREM1 and PDGFRα might also play a role in regulating TIMP1 expression, MEFs were serum-starved overnight and incubated with PDGFCC as described above. Profiling *Timp1* expression in these cells showed that the gene is upregulated by 60 minutes in WT cells, but that the levels in *Frem1^bat/bat^* cells were essentially unchanged in response to PDGFCC (Fig. 3A). Increased phosphorylation of AKT and ERK1/2 by 50 minutes after exposure to growth factor (Fig. 3B) preceded the increases in *Timp1* gene expression and secreted protein levels in WT but not *Frem1^bat/bat^* MEFs (observed at 60 and 120 minutes, respectively) (Fig. 3B,C). The mechanism by which PDGFCC regulates *Timp1* transcription in the dermis is unclear. Studies using EGF as an agonist showed that secretion of both TIMP1 and MMP9 in trophoblasts was downstream of the PI3K and MAPK pathways (Qiu et al., 2004). To investigate this process, WT MEFs were pre-treated with the PI3K inhibitor LY294002, AKT1/2 inhibitor or the MAPK inhibitor UO126 prior to PDGFCC stimulation (Fig. 3D). Treatment of cells with LY294002 or AKT1/2 inhibitor abolished AKT phosphorylation, whereas UO126-treated cells remained unchanged. Phosphorylation of ERK1/2 was ablated in UO126-treated cells, but remained unaffected in LY294002- and AKT1/2-inhibitor-treated groups. Analysis of *Timp1* mRNA expression by qRT-PCR found that inhibition of the PI3K/AKT or MAPK pathways by these compounds blocked *Timp1* mRNA transcription upon PDGFCC stimulation (Fig. 3E). Taken together, these results indicate that a consequence of FREM1 mutation in the developing embryo is a reduction in TIMP1 activation downstream of the PDGF receptor in a pathway mediated by PI3K/AKT and MAPK.

Collagen I (COL1) is a fibrillar collagen that is present in the dermis and basement membrane, and its skin deposition was most recently shown to be specifically regulated by TIMP1 activity (Yokose et al., 2012). In the absence of a reliable antibody to directly detect murine TIMP1, as a proxy for TIMP1 activity *in vivo* we examined basement membrane COL1 deposition in fetal head skin in mice at E13.5, with an antibody to native COL1 (Fig. 3F). We noted firstly that, at this developmental stage, there were significant amounts of COL1 in the basement membrane (in addition to general dermal and epidermal expression) and that FREM1 mutation leads to a specific reduction in the presence of the protein at this site. We quantified the fluorescence intensity of COL1 in the epidermis over keratin 14 (K14), and this confirmed a significant reduction in COL1 in *Frem1 bat* mutants (Fig. 3G). On the basis of our *in vitro* and *in vivo* studies, we propose that the loss of FREM1 has a dual impact on the basement membrane. Firstly, ablation of FREM1 results in a destabilised FRAS-FREM complex, thereby removing a physical cross-link. However, secondly, the loss of FREM1 has a knock-on effect of reducing PDGFRα signal transduction, which lowers expression of *Timp1* and thereby permits MMP-mediated erosion of COLI and potentially other ECM and basement membrane components.

## DISCUSSION

A signature feature of Fraser syndrome and its related diseases is cryptophthalmia, which is thought to arise as a consequence of the formation of large epidermal blisters during embryonic development. Although the identity of the *FRAS* and *FREM* genes mutated in these conditions have been known for many years, the mechanisms by which they mediate epidermal blistering remain unclear. Studies of the blebs mice suggest that the proteins form a mutually stabilising complex that acts at the interface between the basement membrane lamina densa and its underlying dermis, and seems to be crucial for epidermal consolidation. However, our findings suggest that the importance of this complex and the presence of its individual components extend far beyond a simple physical stabilisation. In particular, when considered as a complex, the FREM and FRAS proteins are comprised of nearly 100 recognisable and highly post-translationally modified domains, many of which might be expected to interact with other molecules in the extracellular milieu. Our studies indicate that PDGFCC is one such protein.

**Fig. 4.**
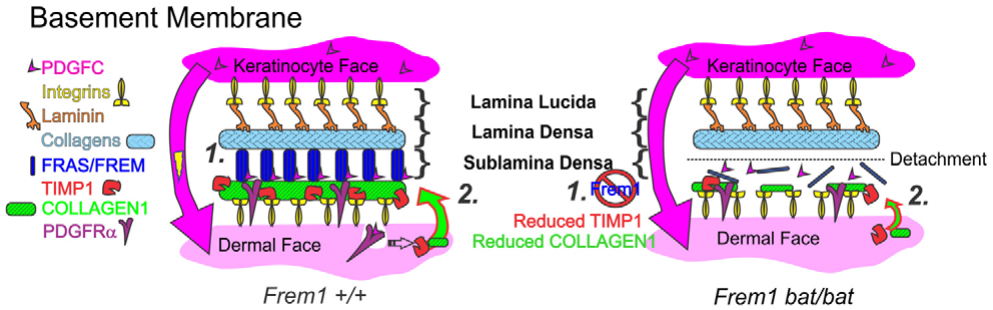
**Summary model of FREM1 *bat* mutant basement membrane fragility.** (Left) In WT mice, FREM1 forms a stabilising complex (1) with FRAS/FREM proteins to cross-link the lamina densa with the underlying dermis. Additionally, FREM1 binds keratinocyte-secreted PDGFC, which is presented to PDGFRα in the adjacent dermal fibroblasts, potentiating PDGFRα signalling and promoting ECM modelling events, including TIMP1 upregulation and COL1 deposition (2). (Right) In *bat* mice, FREM1 mutation removes the structural cross-link of the FRAS/FREM complex (1), but also reduces PDGFRα signalling, leading to lowered TIMP1 and diminished COL1 deposition (2), thereby further weakening part of the foundation of the basement membrane.

During cutaneous development, PDGFC, produced in the epidermis, signals to PDGFRα in the underlying cells of the dermis. We provide evidence that FREM1 binds PDGFC both in culture and in developing embryos (Fig. 1D–G) and colocalises with PDGFC in close proximity to PDGFRα in dermal ECM and the basement membrane (Fig. 1B; Fig. 2D; modelled in Fig. 4). Each domain of FREM1 can separately interact with PDGFC; however, full-length FREM1 does so with the strongest affinity (Fig. 1F,G). The multiple domain interaction of FREM1 to PDGFC is reminiscent of the interaction of PDGF with other ECM components. In the case of NG2 and PDGFA, for example, both the CSPG-containing domain 2 and juxtamembrane domain 3 can bind to PDGFA (Goretzki et al., 1999). Similarly, multiple PDGF-binding sites on perlecan (also known as HSPG-2) contribute to its interactions with this protein (Göhring et al., 1998; Goretzki et al., 1999). The presence of CSPG domains in other basement membrane proteins implies that they might act in a similar fashion to FREM1 and warrants further investigation. This could also explain why no changes in PDGFC distribution were observed in *bat* embryos (Fig. 1B) despite FREM1 mutation.

Our study has demonstrated that the FREM1-PDGFC interaction regulates downstream signalling through PDGFRα to promote TIMP1 expression and thus regulate ECM remodelling. Interestingly, TIMP1 activity regulates COL1 deposition in the skin (Yokose et al., 2012) and transgenic mice overexpressing PDGFC in the liver develop fibrosis with increased COL1 content (Lai et al., 2011), whereas PDGFC and PDGFRα knockout mice develop skin blistering (Soriano, 1997; Tallquist and Soriano, 2003). Here we demonstrate that FREM1 *bat* mutants also exhibit reduced TIMP1 expression and lowered basement membrane COL1 deposition (Fig. 3). We cannot completely exclude the possibility that changes in COL1 deposition in *bat* mutants reflects a non-specific reduction of ECM molecules as a consequence of FRAS1-FREM-complex disruption; however, the reduced COL1 deposition observed is consistent with the associations between COL1 and both PDGFC and TIMP1 reported in the literature (Lai et al., 2011; Yokose et al., 2012). Thus, we propose that FREM1 mutation alters its association with PDGFC and thereby alters the cascade of downstream PDGFRα signalling events, changing the expression profile of proteases and inhibitors (such as TIMP1) to aberrantly impact ECM deposition.

How this interaction more broadly shapes receptor activation remains to be determined; however, we can rule out influences on the proteolytic processing and dimerization of PDGFC. Because this interaction occurs on an ECM scaffold in close proximity of PDGFRα, perhaps WT FREM1 facilitates the presentation of PDGFC to PDGFRα on the surface of dermal fibroblasts contacting the ECM and, in particular, contacting the basement membrane. This model is consistent with our core findings that FREM1 is not absolutely required for PDGFC signalling but instead alters the amplitude and duration. This study provides an additional mechanistic insight into how FREM1 regulates adhesion of the developing epidermis and identifies a key interaction with the PDGFC growth factor. We therefore propose that FREM1 mutation contributes to basement membrane fragility and blistering, in part via reduced PDGFC signalling leading to impaired ECM deposition.

## MATERIALS AND METHODS

### Expression constructs

Truncated *Frem1* constructs expressing the NV, NV-CSPG, CSPG and CalXβ-Lectin domains were amplified from a full-length *Frem1* cDNA [*pcDNA3.1-Frem1-Flag*; a gift from Kiyotoshi Sekiguchi, Osaka University, Japan (Kiyozumi et al., 2006)], subcloned into *pENTR/D-TOPO* and recombined into *pcDNA-DEST40* (Invitrogen, Carlsbad, CA), which had been modified to incorporate a signal sequence from mouse Igκ chain and a triple MYC tag at the N-terminus. A mouse *PdgfC* cDNA (gift from David Loebel, CMRI, Australia) cloned into *pENTR/D-TOPO* and recombined into *pcDNA-Dest40* generated a C-terminal V5 fusion protein.

### Cell culture and transfection

NIH3T3 fibroblasts (ATCC, Manassas, VA) were maintained in DMEM supplemented with 10% foetal calf serum, 100 units/ml penicillin and 0.1% streptomycin, and passaged before reaching confluence. NIH3T3 transfection was performed using lipofectamine LTX (Invitrogen, Carlsbad, CA). Briefly, 1×10^5^ fibroblasts were seeded onto 22×22 mm COLI-coated, 1% BSA blocked coverslips in six-well plates, cultured for 24–48 hours, then each well transfected with a total of 2.5 μg of plasmid DNA (prepared with Qiagen plasmid purification kits), 10 μl of lipofectamine LTX and 2.5 μl of Plus reagent. Wells were provided with fresh media and transfection mix prepared in 0.5 ml Optimem was added with gentle mixing. After 4 hours, wells were washed once with PBS and media replaced. At 48 hours after transfection, fibroblasts were harvested for immunofluorescence analysis.

HEK293 cells were transfected using Fugene HD reagent (Roche, Indianapolis, IN) according to the manufacturer’s instructions. Primary MEFs were harvested from E13.5 embryos as previously described (Ivetac et al., 2005) and maintained to a maximum of five passages. Prior to growth factor stimulation, cells were serum starved overnight. PDGFCC stimulation was performed with 100 ng/ml recombinant protein (R&D Systems, Minneapolis, MN).

### Antibodies to FREM1

Rat monoclonal (clone 17A6) and rabbit polyclonal antibodies to FREM1 were raised against amino acid positions 1500–1637, corresponding to most of the CSPG repeat 11 and a few residues into repeat 12, as described previously (Petrou et al., 2007), using the Monash Antibody Technology Facility (MATF) and Millipore Custom Antibody production facility (Millipore, Temecula, CA), respectively. Both antibodies were validated by western blotting.

### Additional antibodies

Antibodies used and application (immunofluorescence *−IF*, immunoprecipitation *−IP*, western blotting *−WB*): rabbit anti-total AKT #9272 *−WB*, rabbit anti-pAKT (Ser473) 193H12 #4058 *−WB*, rabbit anti-pAKT (Thr308) #9275 *−WB*, rabbit anti-DYKDDDDK (FLAG-tag) #2368 *−IF,* rabbit anti-p44/42 MAPK (ERK1/2) 137F5 #4695 *−WB*, rabbit anti-p-p44/42 MAPK (ERK1/2) D13.14.4E XP #4370 *−WB*, rabbit anti-MYC-tag 71D10 #2278 *−IF*, mouse anti-MYC-tag 9B11 #2276 *−WB,* rat non-immune IgG bs-0293P *−IF* (Bioss Inc., Woburn, MA), rabbit anti-PDGFRα D1E1E #3174 *−IF/IP/WB*, mouse anti-phospho-Y #9411 *−WB* (Cell Signaling Technology, Beverly, MA), mouse anti-total ERK1/2 *−WB* (BD Biosciences, San Jose, CA), mouse anti-collagen type 1 COL-1 C2456 *−IF*, mouse anti-FLAG M2 F3165 *−WB/IF* (Sigma-Aldrich, St Louis, MO), rabbit anti-K14 PRB-155P *−IF* (Covance, Emeryville, CA), rat anti-laminin γ1 A5 MAB1914P *−IF* (Millipore, Billerica, MA), goat anti-TIMP1 AF980 *−WB*, goat anti-PDGFC AF1447 *−WB* (R&D System, Minneapolis, MN), goat anti-PDGFC (C-17) sc-18228 *−IF* (Santa Cruz Biotechnology, Dallas, TX), mouse anti-V5 R960-25 *−IP* (Invitrogen, Carlsbad, CA), goat anti-V5 AB9137 *−IF* (Abcam, Cambridge, UK). Fluorescent secondary antibodies were Molecular Probes (Invitrogen, Carlsbad, CA). Western blot secondary antibodies were from Millipore (Billerica, MA).

### Immunoprecipitation

For immunoprecipitation experiments, supernatants from HEK293 cell lysates were pre-cleared by incubating with protein A- or G- sepharose, incubated with primary antibody overnight and pulled down by the addition of 25 μl 50% slurry of Protein A-sepharose or protein G-sepharose. Washed beads were analysed by SDS-PAGE and western immunoblotting. PDGFRα was immunoprecipitated (Cell Signaling Technology, Beverly, MA) in cells harvested in 20 mM Tris pH 8.0, 100 mM EDTA, 1% Triton X-100, 1 mM Na_3_VO_4_, and protease inhibitors. Endogenous FREM1 was immunoprecipitated using rabbit anti-FREM1 or pre-immune serum from 2 mg of total embryo protein lysate (at E12.5).

### Western blotting

Phospho-antibody blots used antibodies at 1:1000 and were stripped using Gentle Review Buffer (Amresco, Solon, OH) prior to incubation with total-AKT or total-ERK1/2 antibodies, also at 1:1000. For phospho-AKT densitometry analysis, films were scanned and relative signal intensities of phosphorylated AKT levels (normalised over total AKT) from WT MEFs stimulated for 10 minutes with PDGFCC were defined as 1; all remaining samples were standardised relative to this value.

For AKT and MAPK inhibition experiments, serum-starved cells were pre-treated with 50 μM inhibitors of PI3K (LY294002, Sigma-Aldrich, St Louis, MO), MAPK (UO126, Cell Signaling Technology, Beverly, MA), AKT1/2 (AKT1/2 kinase inhibitor, Sigma-Aldrich, St Louis, MO), or equivalent amount of vehicle control DMSO for 1 hour at 37°C, prior to PDGFCC stimulation for 1 hour at 37°C. For phospho-AKT densitometry analysis, relative signal intensities of phosphorylated AKT levels (normalised over total AKT) from unstimulated WT MEFs were defined as 1, and all remaining samples standardised relative to this value.

For TIMP1 secretion analysis, media samples were concentrated tenfold through 10-kDa MWCO Nanosep columns (PALL Corporation, Cheltenham, VIC, Australia) and immunoblotted using goat anti-TIMP1 antibody at 1:1000.

### Immunofluorescence

Cells grown on coverslips were fixed, permeabilised, blocked and incubated with the nuclear stain 40-6-diamidino-2-phenylindole (DAPI) (Sigma-Aldrich, St Louis, MO) and the antibodies indicated following the protocol previously described for C2C12 myoblasts (Cottle et al., 2007). Embryonic tissues for paraffin sections were fixed in 4% paraformaldehyde, with standard xylene dewaxing, and immunostained as described previously (Cottle et al., 2013), except that antigen retrieval was performed with a Dako PT Link according to the manufacturer’s protocol. Staining was performed in a modified blocking buffer consisting of 3% BSA in PBS with one drop of fish scale gelatin per 20 ml. Due to low titre, fluorescent FREM1 detection in paraffin sections using rat monoclonal antibody required tertiary signal amplification first using rabbit anti-rat-Alexa-Fluor-488 followed by donkey anti-rabbit- Alexa-Fluor-488 and donkey anti-rat-Alexa-Fluor-488. Non-immune rat IgG staining was performed in parallel and confirmed that rat anti-FREM1 staining was distinctive. All fluorescent images were acquired on an Olympus Fluoroview 500 confocal scanning microscope and processed using ImageJ.

### Quantitative real-time PCR (qRT-PCR)

RNA was extracted from MEF samples using Trizol reagent (Invitrogen, Carlsbad, CA) and qRT-PCR performed using the TIMP1 primer set 5′-CAACTCGGACCTGGTCATAA-3′ and 5′-ACAGAGGCTTTCCATGACTG-3′ and Power SYBR green PCR master mix (Applied Biosystems, Mulgrave, VIC, Australia). Triplicate samples were run through a standard 2-step reaction using a Stratagene Mx3000 qPCR machine (Agilent Technologies, Santa Clara, CA).

### Mice

All studies were performed using the *Frem1^bat^* ‘*bat*’ mouse allele (Smyth et al., 2004) in accordance with the regulatory statutes set out by the Monash University Animal Welfare Committee and legislation of the Australian and Victorian governments relating to the use of experimental animals. A minimum of three mice per genotype were analysed.

### Statistical analysis

All statistical analysis was performed using the Student’s *t*-test. *P*-values of less than 0.05 were considered significant.
